# From accuracy to patient outcome and cost-effectiveness evaluations of diagnostic tests and biomarkers: an exemplary modelling study

**DOI:** 10.1186/1471-2288-13-12

**Published:** 2013-01-31

**Authors:** Hendrik Koffijberg, Bas van Zaane, Karel GM Moons

**Affiliations:** 1 Julius Centre for Health Sciences and Primary Care, University Medical Centre Utrecht, Mailstop STR 6.131, P.O. Box 85500, 3508, Utrecht, GA, The Netherlands; 2 Division of Perioperative Care and Emergency Medicine, University Medical Centre Utrecht, Utrecht, The Netherlands

**Keywords:** Diagnostic test, Patient outcomes, Cost-effectiveness analysis, Stroke, Cardiac surgery

## Abstract

**Background:**

Proper evaluation of new diagnostic tests is required to reduce overutilization and to limit potential negative health effects and costs related to testing. A decision analytic modelling approach may be worthwhile when a diagnostic randomized controlled trial is not feasible. We demonstrate this by assessing the cost-effectiveness of modified transesophageal echocardiography (TEE) compared with manual palpation for the detection of atherosclerosis in the ascending aorta.

**Methods:**

Based on a previous diagnostic accuracy study, actual Dutch reimbursement data, and evidence from literature we developed a Markov decision analytic model. Cost-effectiveness of modified TEE was assessed for a life time horizon and a health care perspective. Prevalence rates of atherosclerosis were age-dependent and low as well as high rates were applied. Probabilistic sensitivity analysis was applied.

**Results:**

The model synthesized all available evidence on the risk of stroke in cardiac surgery patients. The modified TEE strategy consistently resulted in more adapted surgical procedures and, hence, a lower risk of stroke and a slightly higher number of life-years. With 10% prevalence of atherosclerosis the incremental cost-effectiveness ratio was €4,651 and €481 per quality-adjusted life year in 55-year-old men and women, respectively. In all patients aged 65 years or older the modified TEE strategy was cost saving and resulted in additional health benefits.

**Conclusions:**

Decision analytic modelling to assess the cost-effectiveness of a new diagnostic test based on characteristics, costs and effects of the test itself and of the subsequent treatment options is both feasible and valuable. Our case study on modified TEE suggests that it may reduce the risk of stroke in cardiac surgery patients older than 55 years at acceptable cost-effectiveness levels.

## Background

The number of diagnostics tests and (bio)markers available in medical practice has been increasing rapidly over the past decades and is likely to increase even further given the quest for stratified and personalized medicine. Consequently, there is growing concern about the overutilization of diagnostic tests and markers, the potential negative health consequences due to over testing, and the steeply rising costs for diagnostic testing [1,2]. To avoid these problems proper evaluation of the downstream consequences or benefits, in terms of improved decision making and patient outcomes, as well as on the cost-benefits of diagnostic testing, is required [3-11]. However, the evaluation of diagnostic tests and markers typically stops after quantifying the accuracy of the test or marker as compared to the prevailing reference standard [4-6,8-11]. The consequences of diagnostic testing strategies may be quantified comprehensively in randomized clinical trials (RCT). However, RCTs of diagnostic strategies are not always recommended since they are often too costly and time consuming. They are also considered less feasible given the large number of available diagnostic strategies that can be compared and the consequently required number of patients [4,10,12-15]. Moreover, the limited time frame of a common RCT does not allow assessment of long-term outcomes or risks related to testing (e.g. radiation risk due to imaging tests) and technological evolution of diagnostics may render the RCT results quickly out-dated. Finally, the overwhelming number of new tests and markers entering the market would require a huge number of diagnostic RCTs for which there may not even be enough patients to recruit. These limitations are also apparent from the low number of such diagnostic (test-treatment) RCTs in current literature [15].

Decision analytic and cost-effectiveness modelling is a valid alternative to diagnostic RCTs to quantify the effects of testing on long terms outcomes and costs. Suitable models can synthesize the evidence from different sources, including pre-clinical studies (e.g. evidence on risks), diagnostic test accuracy studies, and therapeutic intervention studies (e.g. evidence on outcomes) [4,10,16-18]. Subsequently, the life course of hypothetical individuals can be simulated while tracking risk factors, complications, quality of life, and costs, to assess the incremental cost-effectiveness of a new diagnostic strategy [17,19-21].

In this paper we illustrate decision analytic modelling using an empirical study to assess the long term health effects and cost-effectiveness of using a new diagnostic test, modified transesophageal echocardiography (TEE) as compared to care as usual in patients undergoing cardiac surgery. This test is used to detect aortic atherosclerosis as early as possible in order to prevent embolic stroke caused by emboli merging from the atherosclerotic ascending aorta during surgery. Rather than discussing the potential value of decision analytic modelling in general we show that the cost-effectiveness of modified TEE can be estimated without performing a diagnostic RCT and present the elements and results of our analysis.

## Methods

### Description of the example study

The incidence of ischemic stroke after cardiac surgery is about 3%, accounting for 10–20% of postoperative deaths [22-25]. As the prevalence of atherosclerosis increases sharply with age from 10% in patients aged 50–60 years to at least 35% in patients older than 75 years, the post-cardiac-surgery stroke rate also increases to 7% in older patients [26,27]. Post-operative stroke is often caused by emboli from dislodged atherosclerotic plaques in the ascending aorta, when it is cannulated or clamped [22,23,28-30]. Knowing the presence and extent of atherosclerosis prior to sternotomy allows changes in surgical strategy reducing or avoiding manipulation of the ascending aorta, and may therefore reduce the post-operative risk of embolic stroke. Hence, rapid and accurate diagnosis of atherosclerosis in the ascending aorta may result in a decreasing number of ischemic strokes after cardiac surgery caused by emboli.

We compared the modified transesophageal echocardiography (TEE) strategy with the manual palpation strategy. Manual palpation is still the most commonly used method for assessing atherosclerosis in the ascending aorta although it is not very sensitive and may be hazardous, i.e. it may dislodge vulnerable atherosclerotic plaques, which in turn may cause stroke, and it can only be used after sternotomy [31]. The modified TEE method was introduced to enhance conventional TEE by visualizing the distal part of the ascending aorta before incision, using an intra-tracheal balloon-catheter (the A-View catheter, Cordatec Inc. Zoersel, Belgium) to create an ultrasound conducting window [32,33]. For both methods epiaortic ultrasound scanning (EU) was used to assess diagnostic accuracy and potential impact on clinical management. Epiaortic ultrasound scanning may be considered the gold standard for detecting ascending aorta atherosclerosis but cannot be applied prior to surgery and is not available in all medical centres [34,35].

### Model description

We used a Markov decision analytic model to assess the differences in long term health benefits and costs of both strategies [19]. In the modified TEE strategy surgery could be adapted due to the presence of atherosclerosis detected pre-sternotomy with modified TEE. In the manual palpation strategy surgery could be adapted due to the presence of atherosclerosis detected with manual palpation after sternotomy.

**Figure 1:**
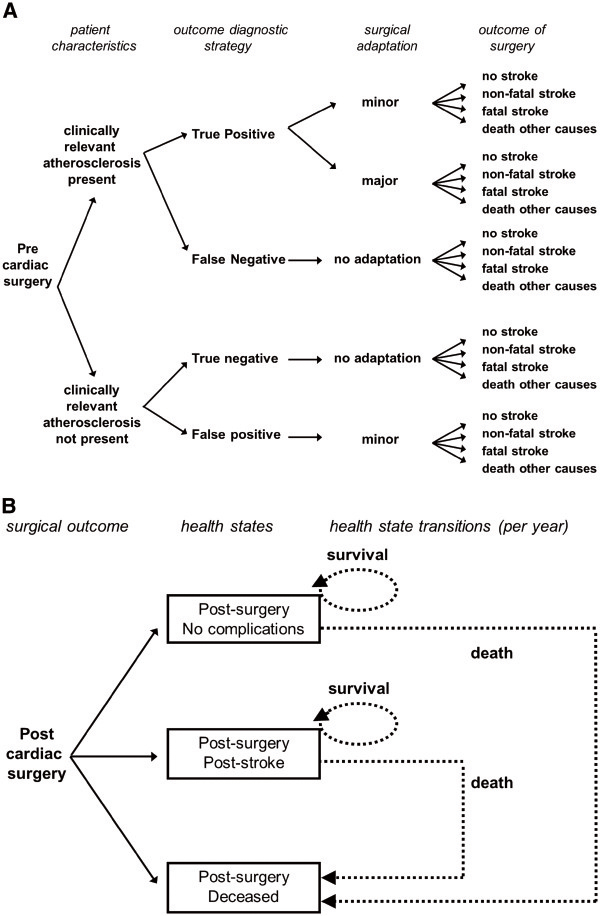
**Visualization of the decision analytic part of the model for the manual palpation strategy [A], and the ensuing Markov part of the model [B].** The structure of the model for the modified TEE strategy is similar.

The decision analytic part of our model (first part) estimated the fraction of cardiac patients that experienced no complications (no stroke), a non-fatal stroke, a fatal stroke, and death due to other causes, separately for each diagnostic strategy (Figure 1A). The outcomes served as input for the Markov model (second part).

Markov models are useful when events can occur at various points in time and with varying probabilities [36]. In a Markov model hypothetical individuals reside in predefined health states and can move from one health state to another in fixed time intervals. We defined the health states ‘post-surgery – no complications’, ‘post-surgery – post stroke’, and ‘dead’, and individuals started in these health states based on the outcome of the decision analytic part of our model (Figure 1B). After a fatal stroke or death due to other causes individuals started, and remained, in the ‘dead’ state. Individuals in the ‘post cardiac surgery – no complications’ state and the ‘post cardiac surgery – post stroke’ state could die from causes unrelated to the surgical procedure, and then move to the ‘dead’ state. Here, the risk of death was based on age and gender specific all-cause Dutch mortality rates (Statistics Netherlands, CBS), apart from the first five years after surgery in which the mortality risk was increased, reflecting the mortality experience of cardiac surgery patients.

We assigned an utility, i.e. a quality of life estimate, and costs to each health state in the Markov part of our model, and also accounted for the costs of diagnosis and adaptation in the decision analytic part of our model.

### The prevalence of atherosclerosis and the risk of stroke

We varied the prevalence of atherosclerosis in our model, allowing it to increase with age, and performed separate analyses for men and women, as accurate data on the age and gender specific prevalence of atherosclerosis is lacking. In total, we defined 12 subgroups based on gender, age (55, 65, 75 year old patients), and two estimates of the prevalence of distal ascending aorta atherosclerosis across age categories. Low prevalence rates were 10%, 20% and 40% at age 55, 65 and 75 years respectively, and high prevalence rates 15%, 30% and 50% respectively. As patients can die from a number of complications arising during or after cardiac surgery, we separated fatal stroke events from fatal non-stroke events. Roughly 15% of all cardiac mortality was attributed to fatal strokes [25]. However, not every stroke needs to be fatal: patients can also experience non-fatal strokes. We included a 38% probability that a stroke is fatal, giving a 62% probability that a stroke is non-fatal [25]. Finally, not all strokes are actually caused by emboli merging from the atherosclerotic ascending aorta, some have other causes and cannot, therefore, be prevented by reducing manipulation of the ascending aorta. In our model, 70% of all strokes were assumed to be embolic and thereby potentially preventable through changes in surgical strategy after detection of atherosclerosis, whereas the remaining 30% was assumed to have other origins.

### Model assumptions

The following assumptions were required for the cost-effectiveness analysis:

1. The beneficial effect from surgical adaptation was similar for minor and for major adaptations (a relative risk of embolic stroke of 0.46 (95%CI: 0.196- 0.910)). However, major adaptations increased surgical costs, whereas minor adaptations (e.g. relocation of clamps) did not. Also, in patients with a false positive test result only minor adaptations may be performed, as any major adaptation is assumed to immediately reveal the incorrect test outcome, and to be aborted as a result.

2. To quantify the expected peri-operative mortality of patients in our model correctly for both strategies, all patients with atherosclerosis in the ascending aorta had the risk factor extra-cardiac arteriopathy as defined in the EuroSCORE risk equation. Consequently, patients with atherosclerosis in the ascending aorta had a higher risk of early mortality than patients without atherosclerosis.

3. The impact of increasing age on quality of life of individuals was assumed to similar for healthy individuals and individuals surviving a stroke. The utility of healthy individuals was therefore set to 1 and the impact of stroke was defined as a reduction in utility from 1 to 0.439, on average.

### Cost-effectiveness analysis

We defined two baseline groups: 65-year old men and 65-year old women, both with a prevalence of 20% atherosclerosis, because these groups contain the largest number of patients seen in clinical practice. For each of the 12 subgroups defined, a hypothetical cohort of 10,000 patients was simulated and the individual pathways of the patients were determined, tracking events, costs, and quality of life, for both diagnostic strategies. Average results were then calculated for each hypothetical cohort. A life time horizon was used, that is, simulation stopped when all patients had moved to the ‘dead’ state, with time intervals (cycles) of one year. We applied a health care perspective. Costs were estimated in euros (1 euro = 1.28 US$, September 10 2012). Costs were discounted with 4.0% and effects with 1.5% according to Dutch guidelines. Based on the difference in health benefits and costs we calculated the incremental cost-effectiveness ratio (ICER). Probabilistic sensitivity analysis was performed by Monte Carlo simulation, and 5,000 simulations were performed to account for parameter uncertainty based on the distributions defined in Table 1. All simulations were performed with TreeAge Pro 2011 software (Williamstown, MA, USA).

**Table 1:** The baseline values for model parameters

**Input parameter**	Value*	**Range$**	**Distribution**	**Source**
Probability of early death (within 30 days)	EuroSCORE risk equation			[37]
Probability death is due to fatal stroke event	13.2%	7.5 – 20.2%	Beta(14,92)	[25]
Probability that a stroke event is not fatal	80.8%	75.3 – 85.8%	Beta(173,41)	[38]
Probability that stroke is caused by emboli	71.0%	63.3 – 77.8%	Beta(107,44)	[38]
Manual palpation: sensitivity	31%	22 – 40%	Beta(33,73)	[31]
Manual palpation: specificity	98%	96 – 100%	Beta(179,4)	[31]
Modified TEE: sensitivity	97%	94 – 100%	Beta(124,4)	[39]
Modified TEE: specificity	80%	74 – 85%	Beta(173,44)	[39]
Risk reduction of post-operative stroke due to adaptations when applying the modified TEE	0.46	0.196 – 0.910	LogNormal (−0.864, 0.392)	[25]†
Probability of a major adaptation of surgery given a patient with atherosclerosis	2.7%	2.3 – 3.1%	Beta(163,5888)	[40]
Risk of death (year 1–2 post-surgery): age < 61 years	1.18%	0.82 – 1.42%	Uniform	[41] ‡
Risk of death (year 1–5 post-surgery): age 61–70 years	2.33%	1.95 – 2.71%	Uniform	[41] ‡
Risk of death (year 1–5 post-surgery): age > 70 years	4.35%	3.49 – 5.24%	Uniform	[41] ‡
Utility of the ‘Post Stroke’ health state	0.439	0.186 – 0.653	Triangular	[42]^
Utility of the ‘No complications’ health state	1	-	-	Default
Cost of death (Euros)	2,736	1,368 – 5,471	Uniform	Expert opinion [43]
Diagnostic cost of modified TEE tool (Euros)	212	184 –243	Gamma	Manufacturer
Cost of fatal stroke event (Euros)	2,736	1,368 – 5,471	Uniform	#
Discount rate for costs	4.0%	-	-	Dutch guidelines
Discount rate for effects	1.5%	-	-	Dutch guidelines

The baseline values that were used as input parameters for the model, along with their range and distribution (if applicable).

* All costs were recalculated to 2012 euros.

$ Range concerns the full range for uniform distributions and 95% confidence intervals for other distribution types.

# The cost of a fatal stroke was assumed equal to the cost of death, as the costs of additional medical care delivered to stroke patients prior to death is already incorporated in the costs of surgery (Additional file 1).

† This risk reduction was based on the comparison of two groups in the original article: group C (13 strokes in 690 patients) versus group A (12 strokes in 268 patients).

‡ Values were recalculated from the actuarial survival curves by age in the source material and concern annual mortality risks applied in the first five years after cardiac surgery.

^ Estimates of the utility of patients after major stroke vary widely therefore a large range was used (min = 0.11, max = 0.71, most likely value = 0.50, resulting in an expected value of 0.439).

## Results

### Pre-existing evidence on the accuracy of the two diagnostic strategies

For manual palpation we found a sensitivity of 31% (95%CI: 22–40%) and a specificity of 98% (95%CI: 96–100%) when compared with EU [31]. For modified TEE a crude sensitivity of 97% (95%CI: 94–100%) and specificity of 80% (95%CI: 74–85%) has been reported [39]. These estimates were used in the model and relate to severe atheroma which, when observed, would require adaptation of the surgical procedure (Table 1). For both diagnostic strategies false positive results could result in minor adaptations, and false negative results caused a lack of adaptations resulting in increased risk of embolic stroke.

### Expected early mortality in cardiac surgical patients

We used the logistic version of the validated EuroSCORE risk equation (comparable with the STS database) to predict early (within 30 days) mortality of the patients in our model [37,44,45]. The prevalence of the risk factors included in the EuroSCORE was derived from the literature [45]. The risks predicted by the EuroSCORE equation are substantially higher than currently observed in the Netherlands, therefore, we reduced the predicted risks for our cohort of patients by 71%, the reduction found in a large study on fast-track anaesthesia in cardiac surgery [41,46].

### Impact of diagnostic tests on surgical management, costs, and the risk of embolic stroke

In the model, application of the modified TEE method was expected to result in major adaptations in 2.7% (95%CI: 2.3-3.1%) of all patients with detected atherosclerosis, similar to the influence of EU on intraoperative surgical management [40]. Half of these adaptations were in patients undergoing coronary artery bypass grafting (CABG) or single valve surgery procedure, and half in complex CABG (> 2 arterial grafts), CABG + single valve or double valve procedure. The adaptations all consisted of ascending aorta replacement with CABG. On patient level, the mean cost of surgery without adaptation equals €15,628, and of surgery with major adaptation €23,886. However, major adaptations occur only in 2.7% of all patients with atherosclerosis, rendering the overall ‘weighted’ mean costs rather similar, i.e. €15,628 without adaptation and €15,842 with the occasional major adaptation (see Additional file 1). In both strategies the detection of atherosclerosis in the remaining 100–2.7% = 97.3% of patients with atherosclerosis resulted in minor surgical adaptations, without cost consequences, such as changing the cannulation site and changing the aortic clamping site. The cost of the modified TEE intervention itself was relatively small (€212,-), as the balloon-catheter is made from inexpensive materials, and the modified TEE imaging procedure takes, on average, only 4.5 minutes to perform [33]. Finally, surgical adaptation was estimated to incur a relative risk of post-operative embolic stroke of 0.46 (95%CI: 0.196-0.910) [25]. Details on costs of cardiac procedures, non-fatal strokes, and potential major changes in surgical procedures are provided in the Additional file 1.

### Cost-effectiveness outcomes

Table 2 presents the intermediate outcomes of the analysis, the predicted mortality risk, the risk of stroke, the probability of adaptation, and the expected number of life years after surgery. As expected, for both the manual palpation strategy and the modified TEE strategy more adaptation occurred with increasing prevalence of atherosclerosis (Table 2). The risk of fatal and non-fatal stroke events also increased with this prevalence and with age, for both strategies. The number of life-years after cardiac surgery decreased rapidly with patient age, but only slightly with increasing prevalence of atherosclerosis, for both strategies. The risk of stroke was higher in women than in men, as female sex is a risk factor in the EuroSCORE risk equation. However, the life-expectancy after surgery was still higher among women. For all patient subgroups, the modified TEE strategy consistently resulted in a larger number of adapted procedures and, consequently, in a lower risk of stroke and a slightly higher number of life-years.

**Table 2:** Expected probabilities of surgical adaptation and stroke

	***Subgroup***					***Manual palpation strategy***					***Modified TEE strategy***			
	Gender	Age	% atherosclerosis	EuroSCORE predictedmortality risk	Chance ofminor adaptation	Chance ofmajor adaptation	Risk of stroke		Life-years after surgery	Chance of minor adaptation	Chance of majoradaptation	Risk of stroke		Life-yearsafter surgery
							non-fatal	fatal				non-fatal	fatal	
1	Men	55 yr	10%	1.04%	3.03%	0.08%	1.01%	0.24%	23.06	9.43%	0.26%	0.84%	0.23%	`23.10
2	Men	55 yr	15%	1.08%	4.54%	0.13%	1.04%	0.24%	23.05	14.14%	0.39%	0.86%	0.23%	23.10
3	Women	55 yr	10%	1.41%	3.04%	0.08%	1.36%	0.32%	26.09	9.43%	0.26%	1.14%	0.31%	26.16
4	Women	55 yr	15%	1.46%	4.54%	0.13%	1.40%	0.33%	26.08	14.14%	0.39%	1.16%	0.31%	26.15
**5**	**Men**	**65 yr**	**20%**	1.61%	6.07%	0.17%	1.53%	0.36%	15.17	18.84%	0.52%	1.25%	0.33%	15.22
6	Men	65 yr	30%	1.71%	9.10%	0.25%	1.61%	0.38%	15.15	28.29%	0.78%	1.29%	0.33%	15.20
**7**	**Women**	**65 yr**	**20%**	2.14%	6.06%	0.17%	2.05%	0.48%	17.49	18.84%	0.52%	1.67%	0.44%	17.56
8	Women	65 yr	30%	2.28%	9.10%	0.25%	2.15%	0.50%	17.44	28.27%	0.78%	1.71%	0.44%	17.53
9	Men	75 yr	40%	3.18%	12.15%	0.34%	2.94%	0.69%	8.73	37.72%	1.04%	2.31%	0.59%	8.80
10	Men	75 yr	50%	3.36%	15.13%	0.42%	3.10%	0.72%	8.70	47.13%	1.31%	2.39%	0.60%	8.77
11	Women	75 yr	40%	4.13%	12.13%	0.34%	3.83%	0.90%	10.44	37.70%	1.04%	3.02%	0.77%	10.55
12	Women	75 yr	50%	4.34%	15.21%	0.42%	4.02%	0.94%	10.40	47.11%	1.31%	3.09%	0.78%	10.53

The probabilities and life-years observed from the model. For both strategies the observed chance of adaptation of the surgical procedure is shown, the observed risk of stroke and the expected number of life-years following surgery. In addition, the EuroSCORE predicted mortality risk is given for each subgroup.

For both strategies costs increased and health effects decreased with increasing patient age, whereas increasing prevalence of atherosclerosis had very limited influence (Table 3). In our main scenario concerning 65 year old men with 20% atherosclerosis manual palpation resulted in costs of € 26,589 to gain 10.75 Quality-adjusted life-years (QALYs), and modified TEE resulted in costs of € 26,581 to gain 10.77 QALYs. In the corresponding scenario for women, costs and effects were € 26,720 and 11.83 QALYs for manual palpation and € 26,662 and 11.86 QALYs for modified TEE, respectively. The modified TEE strategy was slightly more expensive in 55-year-old patients, i.e. subgroups 1–3, and just cost-saving in 65-year-old patients (Table 3). In 75-year-old patients modified TEE offered substantial cost-savings. Cost-savings were higher in women than in men. Furthermore, the modified TEE strategy resulted in small health gains, increasing with age, in all subgroups. Accordingly, the incremental cost-effectiveness ratio (ICER) was positive in subgroup 1–3, indicating additional health at additional cost, with a maximum ICER of €4,651 per QALY. In all other subgroups the ICER was negative indicating additional health at reduced cost. Here the modified TEE strategy dominated the manual palpation strategy.

**Table 3:** Expected costs and effects for the predefined patient subgroups

	***Subgroup***			***Manual palpation strategy***		***Modified TEE strategy***		**Modified TEE vs Manual palpation**		
	Gender	Age	% atherosclerosis	Expectedcosts (€)	Expected effects(QALY)	Expectedcosts(€)	Expectedeffects(QALY)	Expectedincremental costs (€)	Expectedincrementaleffects (QALY)	ExpectedICER(€ / QALY)
1	Men	55 yr	10%	25,896	14.31	25,943	14.32	47	0.01	4,651
2	Men	55 yr	15%	25,936	14.31	25,970	14.32	34	0.01	3,376
3	Women	55 yr	10%	25,984	15.34	25,993	15.36	10	0.02	481
4	Women	55 yr	15%	26,012	15.33	26,009	15.36	−3	0.03	−103*
**5**	**Men**	**65 yr**	**20%**	26,589	10.75	26,581	10.77	−8	0.02	−420*
6	Men	65 yr	30%	26,671	10.74	26,639	10.76	−32	0.02	−1,603*
**7**	**Women**	**65 yr**	**20%**	26,720	11.83	26,662	11.86	−58	0.03	−1,946*
8	Women	65 yr	30%	26,815	11.80	26,722	11.84	−93	0.04	−2,318*
9	Men	75 yr	40%	27,629	7.01	27,479	7.04	−149	0.03	−4,973*
10	Men	75 yr	50%	27,733	6.99	27,547	7.03	−186	0.04	−4,648*
11	Women	75 yr	40%	28,563	8.10	28,182	8.15	−380	0.05	−7,608*
12	Women	75 yr	50%	28,719	8.08	28,264	8.14	−455	0.06	−7,579*

Cost and effect outcomes for each of the 12 predefined patient subgroups (see text). For both strategies the costs and effects are given, as well as the incremental costs and effects and the incremental cost-effectiveness ratio (ICER).

* In this scenario the modified TEE strategy dominated the manual palpation strategy.

**Figure 2:**
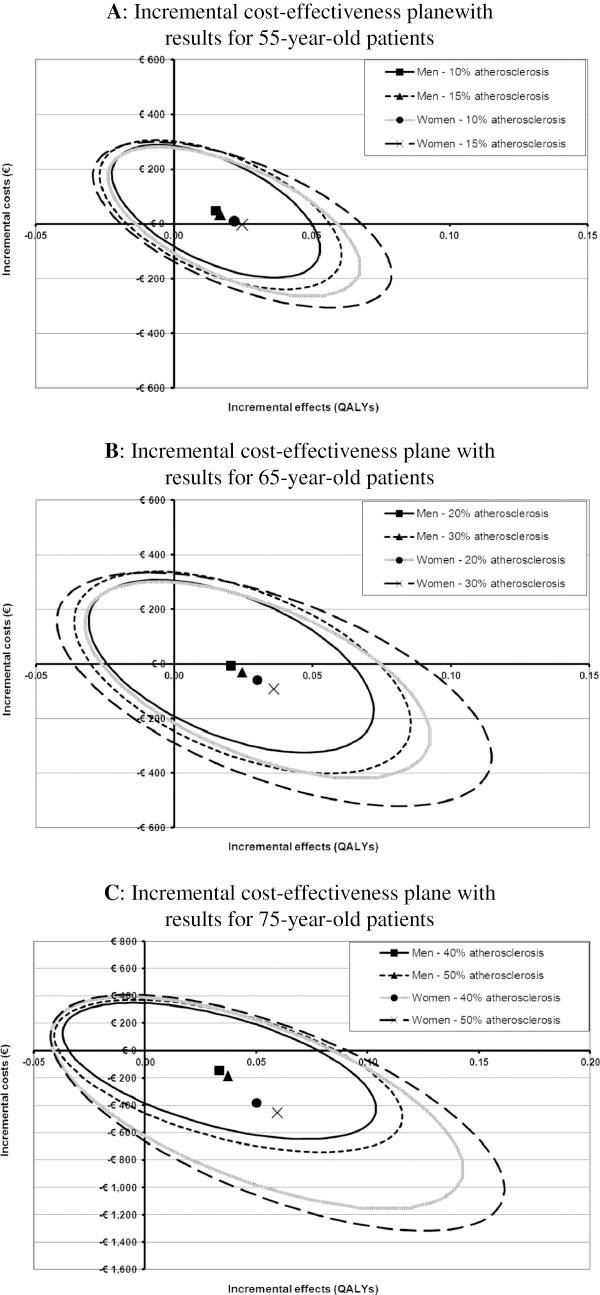
**The cost-effectiveness plane with incremental costs and effects for 55-year old patients [A], 65-year old patients [B], and 75-year old patients [C].** These figures show the incremental effects (x-axis) and incremental costs (y-axis) of the modified TEE strategy. Each of the 12 subgroups is visualized by an ellipsoid that represents the combined bivariate 95% confidence interval (CI) for incremental costs and effects, and a symbol denoting the mean incremental costs and effects. The scales of the axes in Figures 2A-C are not similar, as the range of incremental costs and effects increases with patient age.

Figures 2A-C depict the uncertainty surrounding the cost-effectiveness results shown in Table 3. In these figures the ellipsoids indicate the 95% confidence regions for incremental cost and effects, for different subgroups of patients, based on the results from the probabilistic sensitivity analysis. In all figures the largest proportion of the ellipsoids is located either the north-eastern or south-eastern quadrants of the plane, indicating that the modified TEE is expected to result in health gain. To some extent the ellipsoids also cover the north-western quadrant, indicating increased costs and worse health outcomes for the modified TEE strategy. Comparing men and women in Figures 2A-C it appeared that the uncertainty was consistently larger for women than for men, and increased with the prevalence of atherosclerosis and age. In addition, larger proportions of the ellipsoids were located in the south-eastern quadrant. Thus, the probability of the modified TEE strategy resulting in cost saving and health gain increased although the exact cost-saving and health gain became more uncertain.

**Figure 3:**
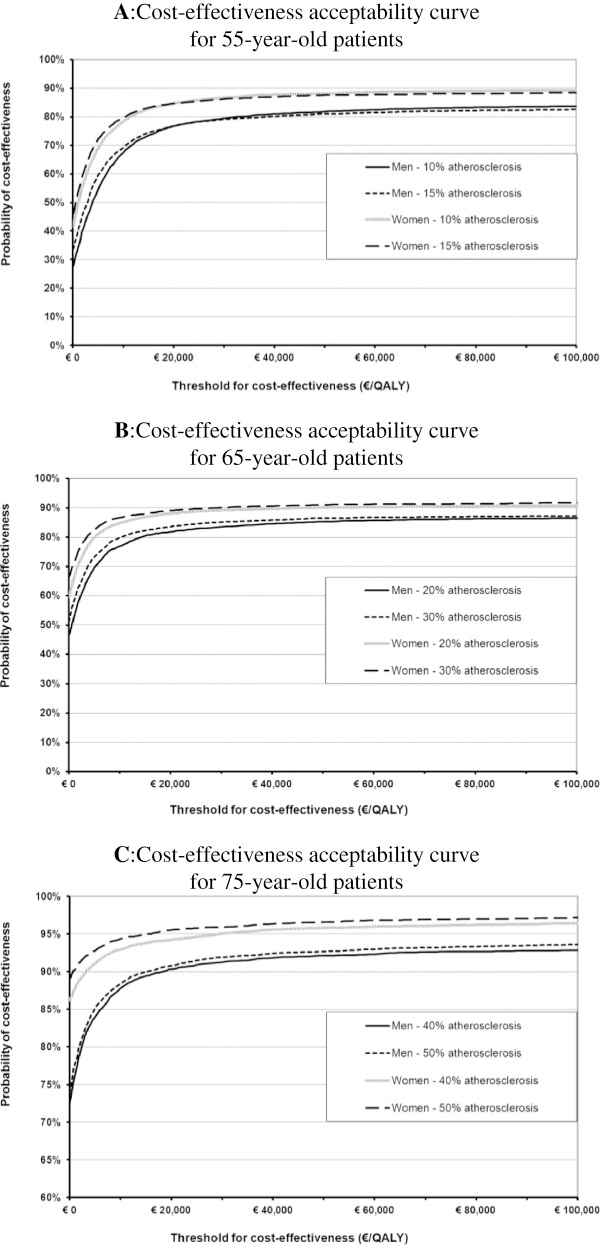
**Cost-effectiveness acceptability curves indicating the probability that the modified TEE strategy is cost-effective for 55-year old patients [A], 65-year old patients [B], and 75-year old patients [C], as function of a range of cost-effectiveness threshold values.** The intersections of the curves with the vertical axis indicate the probability that the modified TEE strategy is cost-saving and results in health gain, i.e. the probability that the cost-effectiveness is still acceptable even if a decision-maker is unwilling to pay anything for additional health gain. The y-axis of Figure 3C was adjusted for visual clarity.

Figures 3A-C shows cost-effectiveness acceptability curves indicating the probability that the cost-effectiveness of the modified TEE strategy would be acceptable for various cost-effectiveness threshold (CET) values [47]. In Figures 3A-C the intersections of the curves with the vertical axis indicate the probability that the modified TEE strategy is cost-saving and results in health gain, i.e. the probability that the cost-effectiveness is still acceptable even if a decision-maker is unwilling to pay anything for additional health gain. Figure 3A shows that for 55-year-old patients this probability ranged from 27% for men (prevalence atherosclerosis 10%) to 46% for women (prevalence atherosclerosis 15%). Figure 3B shows that for 65-year-old patients this ranged from 46% for men (prevalence atherosclerosis 20%) to 66% for women (prevalence atherosclerosis 30%), and for 75-year-old patients this probability ranged from 72% for men (prevalence atherosclerosis 40%) to 89% for women (prevalence atherosclerosis 50%) (Figure 3C). Considering a cost-effectiveness threshold of €20,000/QALY as applied in The Netherlands, the probability that modified TEE has acceptable cost-effectiveness is at least 77% for 55-year-old patients, at least 82% for 65-year-old patients, and at least 90% for 75-year-old patients.

## Discussion

In this paper we demonstrated the value of a comprehensive decision analytic approach in a specific case study on the prevention of post-operative embolic stroke in cardiac surgery patients using different diagnostic test strategies. With respect to our case study it has been shown previously that the modified TEE strategy allows imaging of the atherosclerosis of the distal part of the ascending aorta without severe and unexpected adverse effects [33]. Here we find that the cost-effectiveness of the modified TEE strategy compared with the manual palpation strategy varies with patient age and gender, and with the prevalence of atherosclerosis of the ascending aorta. Nevertheless, our results indicate that, in comparison with manual palpation, the modified TEE strategy is expected to be cost-saving and beneficial in all patients undergoing cardiac surgery from the age of 65 years, also for low prevalence rates of atherosclerosis. In patients 55 to 65 year old, however, the modified TEE strategy is expected to result in small health benefits at very low costs, with a probability of acceptable cost-effectiveness of >77% given the Dutch threshold of €20,000/QALY.

### Limitations of the case study analysis

Our model has certain limitations. First, to obtain an estimate of the risk of death in patients with atherosclerosis in the ascending aorta after cardiac surgery we used the increased risk of patients with arteriopathy as based on the EuroSCORE. This assumption was necessary because accurate evidence on the true excess risk of death due to atherosclerosis in the ascending aorta when undergoing cardiac surgery is lacking. Second, there are no clear estimates of the prevalence of atherosclerosis in the ascending aorta in patients undergoing cardiac surgery, certainly not as a function of patient age. We resolved this issue by defining 12 different patient subgroups with two prevalence rates per age category. We found that the cost-effectiveness results were rather similar for both low and high prevalence rates of atherosclerosis per age category. Finally, the incidence of embolic stroke increases with the complexity of the surgical technique (maximum for combined cardiac surgery and carotid endarterectomy procedures, followed by surgery of the thoracic aorta and combined valve and CABG surgery). However, evidence on the risk of embolic stroke per surgical technique is lacking and could therefore not be included in the analysis.

In the past two decades, diagnostic research methodology has progressed rapidly and in many directions, but important challenges remain [48]. As diagnostic RCTs to assess the long term effects of a new diagnostic test, marker or strategy are often inefficient, infeasible or too time consuming [4,10,12-15], decision analytic and cost-effectiveness modelling can be a valuable alternative. Such approach may synthesize all available evidence to provide insight into the effects of diagnostic strategies, through downstream clinical management, on patient outcomes and costs-effectiveness, both short and long term. Examples of decision analytic models used in cost-effectiveness analysis of diagnostic tests can be found in the literature, but are often limited in their approach [49,50]. For example, they do not include and synthesize relevant evidence on all possible diagnostic pathways, or focus only on short term consequences, or perform only limited sensitivity analysis.

Cost-effectiveness models for evaluating diagnostic tests will, in general, be more complex and require more evidence, than cost-effectiveness models for evaluating therapeutic interventions. However, we showed that cost-effectiveness modelling of diagnostics tests is feasible and can rapidly, at relatively low costs, provide insights into the value of the use or introduction of a new test or marker in terms of improved patient outcomes. Moreover, models can be readily updated when new evidence – e.g. on new treatments or new tests – becomes available. Furthermore, model-based methods can be used to determine whether a diagnostic RCT may still be worthwhile to conduct and in which specific patient groups, to optimize RCT design [19,51].

## Conclusion

Our analysis demonstrates how to use a decision analytic and cost-effectiveness modelling approach to assess the long term cost-effectiveness of a new diagnostic test, marker or strategy - as compared to existing tests or strategies - without doing a formal RCT. The approach combines evidence on the costs and direct risks of the test from early clinical studies, on the performance of the test as compared to the existing reference standard from test accuracy studies, and evidence on the effects of subsequent treatments as obtained from treatment follow-up studies. Our case study suggests that modified transesophageal echocardiography may reduce the risk of embolic stroke in cardiac surgery patients older than 55 years at acceptable cost-effectiveness levels.

## Abbreviations

CABG: Coronary artery bypass grafting;CET: Cost-effectiveness threshold;CI: Confidence interval;EU: Epiaortic ultrasound scanning;ICER: Incremental cost-effectiveness ratio;Modified TEE: Modified transesophageal echocardiography;QALY: Quality-adjusted life-year;RCT: Randomized clinical trial

## Competing interests

The authors declare that they have no competing interests.

## Authors’ contributions

HK developed the decision analytic model, analysed the results and drafted the manuscript. BZ acquired the input data for the model, critically assessed and interpreted the results, and contributed to drafting the manuscript. KGMM conceived the study, participated in interpretation of the results and contributed to drafting the manuscript. All authors have given final approval of the version to be published.

## Pre-publication history

The pre-publication history for this paper can be accessed here:

http://www.biomedcentral.com/1471-2288/13/12/prepub

## Supplementary Material

Additional file 1 Definition of the surgical procedures and cost of stroke. Click here for file
