# Supplementary material for: From accuracy to patient outcome and cost-effectiveness evaluations of diagnostic tests and biomarkers: an exemplary modelling study
Source: BMC Med Res Methodol. 2013 Jan 31;13:12. doi: 10.1186/1471-2288-13-12 (PMC3724486; doi:10.1186/1471-2288-13-12)
Supplement: Additional file 1 — Definition of the surgical procedures and cost of stroke. [file 1471-2288-13-12-S1.doc]

***Appendix to*: From accuracy to patient outcome and cost-effectiveness evaluations of diagnostic tests and biomarkers: an exemplary modelling study**

***Definition of the surgical procedures and cost of stroke***

In this appendix details are provided on the surgical procedures incorporated in the cost-effectiveness model. Appendix – Table 1 shows the mean costs of the predefined surgical procedure types. The possible switches of surgical procedures and the probability of their occurrence, as a result of major adaptation due to detected atherosclerosis, are shown in Appendix – Table 2. Given the probability of major adaptation and the associated changes in costs, separately for each of the original surgical procedures, the cost associated with changing the surgical procedure can be determined. For example, around 62% of all procedures is of type 203 and each of these procedures has a 1.35% probability of being adapted to a type 207 procedure due to detected atherosclerosis of the ascending aorta. Such an adaptation would then cause the costs to increase from €14,332 to €23,886, i.e. an increase of €9,554. The model also included a chance of a minor adaptation. However, such adaptations did not to lead to switches of procedures but only to changing the cannulation site or changing the aortic clamping site.

Appendix I – Table 3 shows the age-specific and gender-specific costs of stroke. These costs are separated into event costs, i.e. costs induced only once immediately following the stroke incident, and recurring, annual costs. For these cost estimates no standard error was available. Therefore, we choose to represent the uncertainty surrounding these cost estimates with gamma distributions having a variance equal to the mean cost estimates.

**Appendix - Table 1.** Details on the cost of the surgical procedures

| Surgical procedure | Description | Mean cost# |
| --- | --- | --- |
| 203 | CABG or single valve | €14,332 |
| 204 | Complex CABG , CABG + single valve, or double valve | €17,564 |
| 205 | CABG + complex valve, Aortic root replacement | €20,108 |
| 206 | CABG + Aortic root replacement or triple valve | €20,959 |
| 207 | Ascending aorta replacement with CABG | €23,886 |

# Costs are based on tariffs from the Dutch Healthcare Authority and recalculated to 2012 Euros and include in-hospital post-surgery care.

CABG = coronary artery bypass grafting.

**Appendix - Table 2.** Details on the probability of surgical adaptations for each initial procedure

|  | Final procedure (after possible adaptation) | | | | | Total initial percentage |
| --- | --- | --- | --- | --- | --- | --- |
| Initial procedure | 203 | 204 | 205 | 206 | 207 |
| 203 | 62.35% |  |  |  | 1.35% | 63.70% |
| 204 |  | 31.95% |  |  | 1.35% | 33.30% |
| 205 |  |  | 1.00% |  |  | 1.00% |
| 206 |  |  |  | 1.00% |  | 1.00% |
| 207 |  |  |  |  | 1.00% | 1.00% |
| Total final percentage | 62.35% | 31.95% | 1.00% | 1.00% | 3.70% | 100.00% |

**Appendix - Table 3.** Details on the cost of stroke as function of age and gender

|  | Incident cost# | | Annual costs after the event# | |
| --- | --- | --- | --- | --- |
| Age at stroke incident (years) | Men | Women | Men | Women |
| 65 | €24,220 | €20,581 | €5,378 | €4,572 |
| 70 | €25,941 | €25,432 | €5,878 | €5,749 |
| 80 | €21,175 | €27,215 | €4,810 | €6,193 |
| 85 | €20,847 | €27,835 | €4,793 | €6,410 |

"# All costs were based on Struijs JN, van Genugten ML, Evers SM, Ament AJ, Baan CA, van den Bos GA: Future costs of stroke in the Netherlands: the impact of stroke services. Int J Technol Assess Health Care 2006, 22:518-524, and recalculated to 2012 Euros.
